# Autophagy Modulation in Cancer: Current Knowledge on Action and Therapy

**DOI:** 10.1155/2018/8023821

**Published:** 2018-01-31

**Authors:** Mija Marinković, Matilda Šprung, Maja Buljubašić, Ivana Novak

**Affiliations:** ^1^School of Medicine, University of Split, Šoltanska 2, 21000 Split, Croatia; ^2^Faculty of Science, University of Split, Ruđera Boškovića 33, 21000 Split, Croatia

## Abstract

In the last two decades, accumulating evidence pointed to the importance of autophagy in various human diseases. As an essential evolutionary catabolic process of cytoplasmatic component digestion, it is generally believed that modulating autophagic activity, through targeting specific regulatory actors in the core autophagy machinery, may impact disease processes. Both autophagy upregulation and downregulation have been found in cancers, suggesting its dual oncogenic and tumor suppressor properties during malignant transformation. Identification of the key autophagy targets is essential for the development of new therapeutic agents. Despite this great potential, no therapies are currently available that specifically focus on autophagy modulation. Although drugs like rapamycin, chloroquine, hydroxychloroquine, and others act as autophagy modulators, they were not originally developed for this purpose. Thus, autophagy may represent a new and promising pharmacologic target for future drug development and therapeutic applications in human diseases. Here, we summarize our current knowledge in regard to the interplay between autophagy and malignancy in the most significant tumor types: pancreatic, breast, hepatocellular, colorectal, and lung cancer, which have been studied in respect to autophagy manipulation as a promising therapeutic strategy. Finally, we present an overview of the most recent advances in therapeutic strategies involving autophagy modulators in cancer.

## 1. Introduction

Autophagy is a cellular degradation or “self-eating” pathway highly conserved throughout all life kingdoms [1]. This quality control mechanism is responsible for the degradation of protein aggregates as well as excessive or damaged organelles whose disintegrated components are later reused during the biosynthesis of new macromolecules [2, 3]. Autophagy plays an important role in maintaining cellular homeostasis and is therefore constitutively active at a basal level in most cell types. However, during different stress conditions, such as those induced by nutrient starvation, organelle damage, accumulation of abnormal proteins, or during development and cell differentiation [4], autophagy is additionally enhanced to meet the cellular needs. This multistep and fine-tuned process is regulated by autophagy- (ATG-) related proteins originally discovered in autophagy-defective yeast mutants [5].

There are three known subtypes of autophagy: macroautophagy, microautophagy, and chaperone-mediated autophagy (CMA). The first type, macroautophagy, is the main autophagy pathway, so the term “autophagy” usually indicates macroautophagy unless otherwise specified. During macroautophagy, a double-membrane structure, phagophore, is formed, which in a selective or nonselective manner engulfs the cytoplasmic cargo destined for degradation. Once the phagophore takes form, it gradually matures and seals, building a closed autophagosome that finally fuses with the lysosome [6] in order to degrade the autophagosome-trapped cargo. Lastly, degradation products are recycled through the cellular anabolic reactions. In contrast, during microautophagy, the lysosomal membrane itself invaginates the cytoplasmic cargo, which is degraded in the lysosomal lumen [7]. In the third type of autophagy, CMA, the chaperone heat shock cognate protein of 70 kDa (HSC70) recognizes soluble cytosolic target proteins containing KFERQ or KFERQ-like sequence motifs, whereupon the target proteins are delivered to the lysosomal lumen through specific interaction between the HSC70 protein complex and the lysosome-associated membrane glycoprotein type 2A (LAMP2A) [8].

Originally, autophagy was thought to be an entirely nonselective process, but current knowledge demonstrates that it is also decidedly selective and that selectivity is mediated by the specific cargo-receptor proteins [9].

## 2. Signaling Pathways Regulating Autophagy

There are at least two major autophagy regulating pathways, ATG5/7-dependent and ATG5/7-independent [10] that were discovered subsequently. Conventional ATG5/7-dependent autophagy is initiated by the Unc-51-like kinase (ULK) complex consisting of several proteins: ULK1/2 (mammalian orthologs of yeast ATG1), FIP200 (FAK-family interacting protein of 200 kDa), ATG13, and ATG101 [11]. Under nonstressed conditions, the mammalian target of rapamycin complex 1 (mTORC1) phosphorylates ULK1/2 thereby inactivating the ULK complex [12]. In contrast, nutrient-sensitive mTORC1 is suppressed under nutrient-limited circumstances, so the ULK complex consequently remains dephosphorylated, hence activated [13]. Once activated, the ULK complex translocates to the phagophore, where it activates the class III phosphatidylinositol 3-kinase (PI3K) complex composed of VPS34 (phosphatidylinositol 3-kinase Vps34), Beclin1, VPS15, and ATG14 proteins [14]. These events lead to autophagosome formation following the extension and closure of the mature autophagosome. Two ubiquitin-like conjugation systems, ATG5-ATG12 and the microtubule-associated protein 1 light chain 3 (LC3) system, are leading regulators of the elongation and closure of the autophagosomal membrane [15–17]. In the ATG5-ATG12 pathway, ATG7 (E1-like enzyme) activates ATG12 that is transferred to ATG10 (E2-like enzyme) to finally conjugate with ATG5. This ATG5-ATG12 complex additionally interacts noncovalently with ATG16L forming a large multimeric (E3-like) complex. The tripartite complex has a function to conjugate LC3 (Atg8 in yeast) to phosphatidylethanolamine (PE) in order to be loaded as a LC3-PE conjugate, known as LC3-II, into the phagophore [18–21]. In research, this lipidated LC3-II protein is often used as a marker for monitoring autophagy progression, since it localizes to both the inner and outer membranes of phagophores and autophagosomes [22, 23].

**Figure 1:**
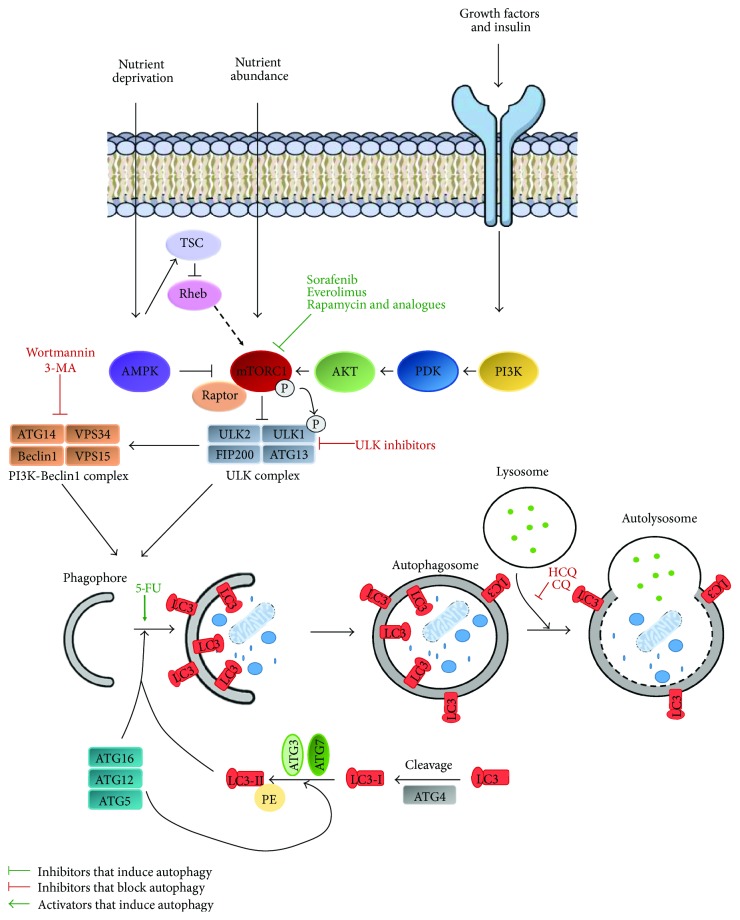
Schematic overview of the autophagy pathway and target points of its modulators. Activation of AMPK and inhibition of mTORC, upon nutrient deprivation, lead to ULK complex activation. Subsequently, ULK complex phosphorylates Beclin1, causing VPS34 activation and phagophore formation. Functional ULK complex consists of ULK1, ULK2, FIP200, and ATG13. VPS34, a regulatory subunit, VPS15, and Beclin1 associate with regulatory factor ATG14 forming functional PI3K-Beclin1 complex. Activation of AMPK inhibits the mTORC complex through the TSC/Rheb pathway. Multiple ATG proteins constitute two ubiquitin-like conjugation systems and mediate the generation of lipidated LC3 proteins, which direct LC3 incorporation into the phagophore membrane. Finally, an elongated phagophore closes, forming autophagosome, which then fuses with lysosome, leading to cargo degradation and nutrient recycling. Current approaches of autophagy modulation are targeting various autophagy steps: activation of autophagy by mTOR complex blockage with sorafenib, everolimus, rapamycin, and its analogues; inhibition of autophagy through inhibition of ULK complex by ULK inhibitors; inhibition of PI3K complex with 3-MA or wortmannin; and activation of autophagy through autophagosome formation induction with 5-FU and autophagosome-lysosome fusion block with HCQ and CQ.

The final step in the degradation process is the fusion of autophagosomes with lysosomes. This process is mediated by three sets of protein families: the Rab GTPases (in autophagy Rab GTPase is Rab7 protein [24, 25]), HOPS—the homotypic fusion and protein sorting-tethering complex [26], and SNAREs—the soluble *N*-ethylmaleimide-sensitive factor attachment protein receptors. Upon starvation in mammals, three SNARE proteins, including syntaxin 17 (STX17), synaptosomal-associated protein 29 (SNAP29), and vesicle-associated membrane protein 8 (VAMP8), mediate autophagosome-lysosome fusion [27, 28] (Figure 1).

ATG5/7-independent autophagy was discovered in 2009 by Nishida et al. [10]. They named it an “alternative autophagy,” since ATG5 and ATG7 were until then considered crucial for mammalian autophagy [10, 29, 30]. Their main observation was that etoposide treatment of the ATG5-deficient MEFs induces autophagy to the same level as in the wild-type cells. Further, it was explained that the ULK1 complex, Beclin1, and PI3K also play a pivotal role as in conventional autophagy. In addition, they demonstrated that upon silencing the ATG5-ATG12 pathway, this did not affect the alternative autophagy, where in turn conventional lipidation of LC3 was replaced with Rab9 activity to control the extension of the phagophores [10]. Therefore, Rab9, which normally mediates transport of proteins from the late endosome to the trans-Golgi membrane [31, 32], was proposed to act in the extension and closure of phagophores in the alternative autophagy that matches the role of ATG5/ATG7/LC3 in the conventional autophagy [3]. Unlike the multiple origin of phagophores in the ATG5/7-dependent autophagy [33, 34], in an alternative autophagy trans-Golgi cisternae seem to be the only membrane source [10].

## 3. Autophagy Modulation as a Promising Therapeutic Target

Autophagy impairments are root causes of numerous diseases such as cancer, neurodegenerative disorders (Alzheimer disease, Parkinson disease), infectious and inflammatory diseases (Crohn's disease), diabetes, obesity, and cardiovascular and muscular diseases [35]. Therefore, the number of studies focusing on the autophagy modulation as a perspective and promising therapeutic target is constantly increasing. Some autophagy modulators, like rapamycin and its water-soluble derivatives (temsirolimus and everolimus), are already being used in cancer therapies. In 2007 and 2009, respectively, temsirolimus and everolimus were approved by the Food and Drug Administration (FDA) for the treatment of advanced renal carcinoma [36, 37]. In 2011, the FDA also approved everolimus for patients with progressive neuroendocrine tumors of pancreatic origin (PNET) [38] and for the treatment of advanced hormone receptor-positive and HER2-negative breast cancers in combination with exemestane [39]. Moreover, in 2012, the European Union approved the use of temsirolimus monotherapy for the treatment of relapsed and/or refractory mantle cell lymphoma [40].

Galluzzi summarized specific autophagy modulators and their current status in the preclinical studies and clinical trials [41]. Here, we present a summarized overview of the most prominent autophagy modulators (Figure 1).

Autophagy activators can be divided into several groups: starvation inducers, endoplasmic reticulum stress inducers, rapamycin and its derivatives, small molecule enhancers of rapamycin, trehalose, inositol monophosphatase inhibitors (IMPase), class I PI3K inhibitors, and other activators [42]. Many of them are in preclinical studies or clinical trials, and several have already been approved for clinical use. The mTORC1 inhibitors, everolimus, and temsirolimus have been approved and are currently being used in cancer therapies, while rapamycin (sirolimus) is used in coronary stents and in rare pulmonary diseases [41, 43, 44]. Drugs such as metformin (for type 2 diabetes treatment) and retinoic acid are also approved for cancer treatment although their modes of action are still unclear [45].

Autophagy inhibitors include PI3K inhibitors, cycloheximide, vacuolar-type H(+)-ATPase inhibitors, lysosomal lumen alkalizers, and acid protease inhibitors [42]. Chloroquine (CQ) and hydroxychloroquine (HCQ) are both lysosomal inhibitors previously used for the prevention and treatment of some types of malaria [46]. Another unconventional drug, azithromycin, a macrolide antibiotic often used for treatment of multiple bacterial infections, was discovered as an autophagy inhibitor after usage in cystic fibrosis patients as an anti-inflammatory drug. Azithromycin prevents lysosomal acidification thereby blocking autophagic degradation [47]. Most of the autophagy inhibitors are still in the preclinical development stages, so we can expect their increased usage in treatment therapies in the upcoming years.

The most common approach in modulating autophagy is the targeting of two different autophagy regulation pathways: 5′ adenosine monophosphate-activated protein kinase (AMPK) and mTOR pathways [48]. The AMPK is a cellular energy sensor that is activated during stress or under scarce glucose conditions [49]. Activated AMPK induces autophagy through different pathways; hence, it is a major regulator of autophagy. This is achieved through AMPK phosphorylation of the ULK1 and TSC1/TSC2 complex that consequently acts on mTORC1 activation as we described earlier [50, 51]. AMPK also plays a very important role in selective mitochondrial autophagy (mitophagy) and mitochondrial biogenesis [50]. Further, it is responsible for the activation of peroxisome (proliferator-activated) receptor gamma coactivator 1-alpha (PGC1-*α*) that regulates the expression of mitochondrial proteins [52, 53]. The second target, mTOR, is a cellular nutrient, energy, and oxygen sensor kinase that regulates cell growth, survival, proliferation, and autophagy in mammals [54, 55]. mTOR kinase belongs to the PI3K-related kinase family and is the main component of the two complexes, mTOR complex 1 (mTORC1) and mTOR complex 2 (mTORC2) [56]. mTORC1 blocks autophagy and controls protein synthesis, lipid biogenesis, and cell growth [48]. Regulation of cell proliferation is controlled by mTORC2 and is crucial for cell survival and maintenance of the actin cytoskeleton [48, 57]. Dysregulation of the mTOR pathway is often correlated with cancer, neurodegenerative, cardiovascular, and renal diseases, and for this reason makes it an ideal therapeutic target [58].

## 4. Autophagy and Cancer

According to the latest predictions of the American Cancer Society, more than 1.6 million new cancer cases are expected to be diagnosed in 2017 in the US. The predictions are really worrying and signify that every third person affected will die from one or the other type of cancer, which is confirmed by the fact that there are about 1600 cancer deaths per day in the US [59]. The reason for these alarming statistics is the fact that carcinogenesis is one of the most complex phenomenon in the evolution of multicellular organisms. Tumor complexity causing tumor heterogeneity, which is manifested not only in different tumor types but also in the cells within an individual tumor, can enormously vary. Amazingly, every single cell within the tumor has the ability to change as the tumor progresses over time, making cancer incredibly difficult to treat. In the last two decades, we have witnessed unimaginable scientific advances in the field of molecular mechanisms underlying autophagy and have learned the importance of autophagy in physiology and disease development. Even in physiological conditions, a low level of basal autophagy is constitutively present which acts as a control mechanism of the cell and provides proper quality and quantity control of the intracellular components. The identification of the Atg genes and their products, discoveries of the molecular mechanisms [60], and the numerous loss-of-function studies on different model organisms have enabled us to understand the role of autophagy and its role in the development, differentiation [4, 61], metabolism [62], immunity [63], and aging [64].

Considering the fact that autophagy is implicated in many different cellular processes, and also keeping in mind the complexity of the molecular mechanisms of tumor initiation and development, it is not surprising that the disturbance of autophagy was found to be one of the possible causes of tumor formation and progression. Indeed, cancer was the first disease connected with disturbed autophagy [65] as well as the first for which clinical trials in humans were performed [66].

Because of the sensitive multistepped nature and complex regulatory processes, autophagy can be disrupted at any stage, which can lead to the development of a whole range of pathologies [67–69]. Therefore, targeting autophagy could be a potential strategy for the treatment of multiple diseases. Considering the dual role of autophagy in cytoprotection and cell death, it is vital to study how autophagy affects a particular type of disease in order to change its modulation in the right direction for the creation of a successful therapy.

### 4.1. Autophagy as a Tumor-Suppressor Mechanism

As mentioned above, autophagy plays a complex dual role in tumorigenesis and is consequently the reason why development of the autophagy-based cancer therapy is so demanding. The preliminary molecular mechanisms of tumor initiation and progression are much more complex than the mechanism of the actual disease development. The basis for any malignant cell transformation is the activation of a protooncogene or the inactivation of tumor suppressor genes. However, a large number of studies confirm that cancer cells also have altered core autophagy regulators, where either their expression levels or genetic information is altered. Knowing this, autophagy could behave similarly as a tumor suppressor (acting to prevent tumor initiation) or as a tumor promoter to ensure tumor longevity via apoptosis inhibition.

The first studies in the 1990s pointed to the relationship between autophagy and tumorigenesis and showed that about 50% of prostate, breast, and ovarian cancers have an absence of one Beclin1 allele [70–72] that codes for Beclin1, a key component in the autophagosome nucleation. Due to haploinsufficiency of the tumor suppressor Beclin1 gene, the level of Beclin1 is reduced compared to the healthy tissue, which consequently suppresses autophagy and causes cancer progression. So far, reduced Beclin1 expression has been confirmed in numerous cancers including cervical squamous cell carcinomas [73], hepatocellular carcinomas [74], osteosarcomas [75], and glioblastomas [76]. Interestingly, some studies have shown that Beclin1 gene expression is increased in stage IIIB colon cancer [77] or in non-Hodgkin lymphomas [78], which stresses the additional protooncogenic role of Beclin1.

Heterozygous deletion of several other core autophagy genes is reported to promote a tumor-suppressor role of autophagy in cancer. Monoallelic deficiency of UV radiation resistance-associated gene (UVRAG), a positive Beclin1/PI(3)K complex regulator, inhibits autophagy and contributes to the emergence of human colon [79] and gastric cancers [80]. Another UVRAG-Beclin1 complex interactor, Bax-interacting factor-1 (Bif-1), was found deleted in gastric and prostate cancers [81]. Since the core signaling pathway of the autophagosome formation is a cascade of amino acids, TORC1-ULK1-VPS34-Beclin1, nutrient starvation, and energy deficiency, common to many developing tumors, lead to mTORC1 inhibition and reactivation of ULK1 kinase activity. This activation phosphorylates Beclin1 on Ser14 and initiates the proautophagy VPS34 complexes to promote autophagy induction and maturation. Tang et al. have stressed that ULK1 can be used as a novel prognostic biomarker for breast cancer after they have found that decreased ULK1 expression is associated with cancer progression [82]. In contrast, an increased ULK1 expression observed in esophageal squamous cell carcinoma [83], hepatocellular carcinomas [84], nasopharyngeal carcinoma [85], and the latest in human gastric cancer [86] is a further indication of the dual role of autophagy as both a tumor suppressor and tumor promoter in cancer. Taking together, these data reveal potential strategic goals in cancer therapy.

#### 4.2. Autophagy as a Tumor-Promoter Mechanism

While autophagy has a tumor-suppressing role in the early stage of carcinogenesis, in advanced cancers, it often acts as a tumor survival or even tumor promoter mechanism. This is mostly due to the fact that tumor cells are resistant to extremely stressful conditions, that is, nutrient and oxygen deprivation, within the tumor tissue. These conditions are even more rigorous in the central part of the solid tumors where the autophagy level is significantly higher than on the periphery [87]. This suggests that autophagy in some tumors also acts as an adaptive mechanism enabling their advancement in the absence of the key survival factors. Yang et al. found that an increased autophagy level in mouse pancreatic cancer led to tumor regression and prolonged lifespan [88]. Another support of the theory comes from studies where the knockouts of core autophagy proteins, ATG5, ATG7, or FIP200, were analyzed. Wei et al. analyzed and reported that with the removal of FIP200 in human breast cancer mouse models, tumor initiation and progression was suppressed [89]. The analysis of multiple cancers showed the overexpression of ATG5 in gastric [90] and prostate [91] cancers while overexpression of ATG7 was seen in bladder cancer [92]. In contrast, mice with systemic mosaic deletion of Atg5 or Atg7 developed benign liver adenomas that do not progress to adenocarcinoma or metastasize [93]. Taken together, these results demonstrate the involvement of core autophagy proteins in tumor development and progression.

In conclusion, depending on the type of tumor and its developmental stage, activation or inactivation of autophagy can contribute differently to tumorigenesis. Reduced autophagy can contribute to tumor progression, whereas increased autophagy may be a mechanism for tumor survival under hypoxic, metabolic, or therapeutic stress conditions. Thus, the modulation of the autophagy process is a promising, but complex, therapeutic strategy for the enhancement of anticancer treatments. A better understanding of the autophagy in tumor models is crucial in identifying new and effective therapeutic strategies for cancer treatment. Next, we summarize the preclinical and clinical usage of autophagy modulators in common cancer types.

Here, we outline a brief overview of current knowledge on modifications of the core autophagy machinery in pancreatic, breast, hepatocellular, colorectal, and lung cancers that represent a promising strategy for the future of drug development. Currently, these tumors represent an example of the successful application of autophagy modulation in preclinical models, which proved to be valuable for novel clinical trials. The chosen cancer types represent an example where the dual role of autophagy, both tumor promoter and tumor suppressor, has been established (Table 1). Moreover, depending on the autophagy role in cancer development and progression, specific preclinical tumor models have been designed specifically aimed at activating or to inhibiting autophagy. Most recent studies on autophagy inhibition have reported on the use of late-stage autophagy inhibitors, CQ or HCQ, which effectively inhibit autophagosome-lysosome fusion. However, their usage as autophagy inhibitors in cancer treatment is quite controversial. Current data proposes that the inhibition of late autophagy by CQ or HCQ might not be the only mechanism of their action in cancer [94–97]. Hence, they can affect tumor cell survivability through the inhibition of immune cell action against tumor cells [97] or influence the permeabilization of the lysosomal membrane thus affecting apoptosis [95]. CQ is often cytotoxic at high doses and can promote cell cycle arrest [96] or DNA damage that induces cancerogenesis [94]. In addition to studies listed in Table 1, results from preclinical studies and early-stage clinical trials with HCQ and CQ in cancer treatments have been reported for some other types of cancers.

According to http://clinicaltrials.gov, there are several ongoing phase I/II trials evaluating the combination of HCQ or CQ with chemotherapeutic agents in patients with multiple myeloma, brain, kidney, prostate, or lung cancer.

### 4.3. Pancreatic Cancer

Pancreatic cancer is the ninth leading cause of cancer deaths worldwide [59]. The most frequent form of pancreatic cancer (almost 85%) are pancreatic ductal adenocarcinomas (PDACs) which are one of the most lethal cancers worldwide [59]. The five-year survival rate is about 4% due to the fact that most PDAC patients are diagnosed with the advanced form of the cancer that has already metastasized and displays a very weak response to currently available therapies [98, 99]. There are several studies confirming the protumorigenic role of autophagy in PDAC carcinogenesis.

Autophagy inhibition with CQ or genetic manipulations by siRNA showed a positive tumor regression response in PDAC models [88]. Activating KRAS mutations were found in over 90% of PDAC [100] that further confirms the critical role of KRAS in PDAC carcinogenesis that came from mouse models [101, 102]. Additional p53 tumor suppressor gene mutations and the loss of heterozygosity have been found in ~75% of PDAC cases that contribute to tumor progression [103]. To study the impact of autophagy deficiency on PDAC, Yang et al. generated a KRAS-driven pancreatic cancer model with conditional heterozygous deletions of p53 and Atg5 alleles. Autophagy inactivation in these mice promoted the formation of premalignant pancreatic intraepithelial neoplasia (PanIN) lesions, but defected autophagy simultaneously prevented their malignant transformation to PDAC [104]. A similar study was performed by Rosenfeldt et al. using KRAS-driven PDAC models with hemizygous Atg5/Atg7 deletion and with and without the p53 mutation [105]. Mice with normal p53 expression and lower ATG5/ATG7 expression accumulated PanIN lesions that did not progress to high-grade PanIN and PDAC indicating the tumor-promoting role of autophagy in this model. In contrast, in the model without p53 and with partial Atg5/Atg7 deletion, which reduces autophagy, tumor formation was shown to be accelerated probably due to the absence of both copies of p53. The authors concluded that in the p53-mutant model, autophagy is not actively promoted. However, the question that remains unanswered is why alterations in the p53-mutant model modify the role of autophagy in PDAC carcinogenesis and tumor progression? Interestingly, although the last mentioned model showed promising results, there is no clinical trial designed to investigate the expression status of p53 and Kras genes together [105] while presently there are six clinical trials that investigate the effect of HCQ on PDAC. The first one, “Study of Pre-surgery Gemcitabine + Hydroxychloroquine (GcHc) in Stage IIb or III Adenocarcinoma of the Pancreas,” examined the effect of p53 mutation status on disease-free period and their overall survivability (NCT01128296, Table 1). The results confirm the data from previous preclinical studies where p53 mutations and autophagy inactivation contributed to poor prognosis of PDAC patients, shortening the disease-free time. Another study is “A Phase I/II Pharmacodynamic Study of Hydroxychloroquine in Combination with Gemcitabine/Abraxane to Inhibit Autophagy in Pancreatic Cancer,” which is currently in the recruiting phase. The endpoints of this trial will be directed to the pharmacokinetics of HCQ considering KRAS genetic status (NCT01506973, Table 1). Although it is expected that most patients with the p53 gene mutation or heterozygous deletion will also have activating KRAS mutations (known presence in more than 90% PDAC patients), the clinical trial is not examining the relationship between the expression status of p53 and KRAS. Therefore, future clinical trials should investigate the p53-KRAS interplay to ensure a proper therapeutic target and strategy in the PDAC treatment development.

Current clinical trials have shown that autophagy inhibition by HCQ as a monotherapy is not sufficient [106]. However, five clinical trials where the combination of autophagy inhibition with DNA synthesis (gemcitabine and capecitabine) or cell division (abraxane) inhibitors are used are still in progress and the results are highly anticipated (NCT01128296, NCT01506973, NCT01494155, NCT01978184, and NCT03344172; Table 1). One possible explanation as to why the combined therapies work better than HCQ monotherapy is because the autophagy could be required to degrade harmful material generated as a result of chemotherapeutic drug insults to the cancer cells. The other reason could be because the inhibition of a single possible pathway is simply not sufficient.

### 4.4. Breast Cancer

The first evidence of how genetic inactivation of autophagy can contribute to the malignant transformation in breast cancer was made by Liang and colleagues in 1999 [107]. They showed that Beclin1 expression is frequently low in human breast epithelial carcinoma cell lines and tissues, but expressed at high levels in normal breast epithelia. Further, they noted how Beclin1 in MCF7 breast carcinoma cells has an autophagy-promoting activity. These findings suggested that the decreased expression of autophagic protein Beclin1 might contribute to the development or progression of breast cancer.

Since 2003, several studies have been performed on genetically engineered breast cancer mouse models with impaired autophagy. Qu et al. and Yue et al. have used a Beclin1 heterozygous mouse model to test whether monoallelic deletion of Beclin1 promotes breast cancer tumorigenesis [108, 109]. This further evidenced how genetic inactivation of autophagy can contribute to a malignant transformation.

A study by Wei et al. revealed that FIP200 as a potential target for cancer therapy since FIP200 ablation in mice, and consequently autophagy inhibition, suppressed mammary tumor initiation and progression [89]. p62/SQSTM1 (known as sequestosome-1, here referred to as p62) is a selective autophagy receptor that binds LC3 and recruits the selected cargo to the maturing autophagosome [110, 111]. The disruption of essential genes within the autophagy pathway, including FIP200, impairs autophagosome biogenesis at the earliest stages and leads to the accumulation of substrates such as p62. As it interacts with a number of proteins in different intracellular signaling pathways, p62 plays an important role at the crossroads of autophagy, apoptosis, and cancer [112–114]. Several studies [115, 116] showed that p62 has a role in protumorigenesis, and Mathew et al. [117] found that p62 accumulation, upon autophagy inhibition in apoptosis-deficient cells, increased tumorigenesis through increased oxidative stress and deregulation of NF-*κ*B signaling. Wei et al. have demonstrated that p62 knockdown or p62 deficiency in already established FIP200-null tumors dramatically reduced tumor growth [118]. Therefore, this later model demonstrated that p62 impairment and suppression of autophagy by FIP200 deletion could synergize to inhibit tumor growth, suggesting new insights for the future design of anticancer drugs.

A study from 2010 revealed a significant association between Beclin1 deletion and human epidermal growth factor receptor 2 (ErbB2) amplification [119], thus providing evidence for decreased Beclin1 expression in a particular breast cancer subtype [120]. A recently established mouse tumor model made by Lozy et al. was the first link between Beclin1 heterozygosity and ErbB2-driven mammary tumorigenesis [121]. This model showed that ErbB2-driven cancers were associated with autophagy suppression. With the proposal of a new model for the PALB2- (partner and localizer of BRCA2) associated hereditary breast cancer, Huo et al. directly demonstrated a tumor-promoting role of autophagy in breast cancer development [122]. Under normal conditions, PALB2 functions as a tumor suppressor similarly to BRCA1 and BRCA2 in maintaining the genome stability and cellular homeostasis. Due to the monoallelic deletion of Beclin1, impaired autophagy results in reduced PALB2-associated breast tumorigenesis in the wild-type p53, but not in a conditionally null background, indicating that Beclin1-related autophagy plays a protumorigenic role in the wild-type p53 PALB2-associated breast cancer, but not in the p53 null tumors.

Currently, several clinical trials are testing the therapeutic potential of different autophagy inhibitors that are used alone or in combination with chemotherapeutic agents. Phase I/II clinical trial was performed to investigate the role of autophagy inhibition on metastatic breast cancer patients using a combined treatment of HCQ and ixabepilone, a chemotherapeutic agent that stabilizes microtubules (NCT00765765, Table 1). The aim of this clinical trial was to show a decrease in tumor growth and a higher tumor response compared to ixabepilone chemotherapy alone. Unfortunately, due to slow patient accrual, the study could not be completed (the main reason for the low accrual was too stringent exclusion criteria). Two clinical trials testing CQ are currently in the recruitment phase. The first one, a phase II study (NCT01446016, Table 1), is testing the effect of CQ in combination with taxane or taxane-like chemotherapy in metastatic breast cancer patients who have previously failed anthracycline chemotherapy. The second, a phase I/II study (NCT01023477, Table 1), investigates the reduction of ductal carcinoma in situ (DCIS) after CQ administration. Yet another phase II trial examines CQ effect on breast tumor cell proliferation and apoptosis (NCT02333890, Table 1). The point of this study is to determine whether CQ will prevent breast cancer growth in patients currently not being treated with neoadjuvant chemotherapy prior to surgical intervention. To conclude, due to the high heterogeneity of this type of cancer, it might be difficult to find unique treatments, making breast cancer a good candidate for a more personalized approach.

### 4.5. Hepatocellular Cancer

Incidence of the liver cancer has been more than tripled from 1980 onwards. A majority of primary liver cancers (75–90%) are hepatocellular carcinomas (HCCs), a carcinoma with very high malignant potential, high recurrence rate, and poor patient prognosis. In the early stage, surgical resection or liver transplantation are proven to be successful. However, for patients at the advanced HCC stage, effective therapy is currently unavailable resulting in low overall survival rates. Autophagy plays multiple roles in maintaining liver homeostasis. In the absence of key autophagy genes, Atg5 and Atg7, nonfunctional proteins and organelles accumulate in liver cells [93]. Analysis of conditional Atg7 knockout mice revealed that these mice develop hepatomegaly and different metabolic liver disorders [30]. HCC is one of the best examples of dual autophagy role in tumors. In the early stage of HCC, during hepatocyte dysplasia, an antitumor role applies. Autophagy contributes to the preservation of the genome stability and the prevention of a malignant transformation by removing harmful mitochondria and transformed cells. A rat model study demonstrated that in the established HCC treatment with CQ, autophagy inhibition acquired a protumor role providing nutrients to HCC cells in the tumor microenvironment and promoted enhanced tumor growth [123]. The tumor-suppressing role of autophagy in HCC has been confirmed on several HCC models. One of the first evidence supporting the tumor suppressor role of autophagy in the cancer formation comes from Beclin1 knockout mice. Biallelic Beclin1 deletion reduces autophagy activity and such mice were more likely to develop cancer, including HCC [108]. In addition, the heterogenic deletion of the Beclin1 reduced autophagy, increasing cell proliferation and initiating spontaneous formations of malignant lesions [108, 109]. It is also interesting to note the correlation between the decreased expression of Beclin1 and HCC grade [74]. This unfolds the possibility to use the Beclin1 gene as a HCC prognostic biomarker [74]. Takamura et al. [93] reported that mice with systemic mosaic deletion of Atg5 exclusively develop benign liver adenomas. These tumors show disordered autophagy features such as mitochondrial swelling, p62 accumulation, oxidative stress, and genomic damage responses. Liver-specific Atg7-deficient mice also developed liver tumors that were reduced in size after p62 deletion [93]. This suggests two things, that autophagy is important for the suppression of tumorigenesis in the liver and that the accumulation of p62 caused by autophagy deficiency contributes to tumor progression.

The latest insight about the antitumor role of autophagy was given by Chen and colleagues [124] who demonstrated that the long noncoding RNAs (lncRNAs) have a regulatory role in HCC. In healthy cells, an elevated expression of the phosphatase and tensin homolog (PTEN) and its homolog PTENP1 results in the PI3K/AKT pathway inhibition. This consequently suppresses the cell proliferation and induces autophagy and apoptosis. As opposed to this, Chen and colleagues have found that the expression of PTEN and PTENP1 is downregulated in several HCC cell lines. However, they have shown that the PTENP1 activity could be restored by lncRNAs or miRNAs. The mentioned miRNAs can also increase the expression of autophagy genes including ULK1, ATG7, or p62, trigger autophagy, and suppress HCC tumor growth. This points to the possibility of using precisely targeted RNA in HCC therapy, but probably in other tumors too.

Cancer cells use autophagy to ensure an alternative energy source for growth and survival in a stressful tumor microenvironment with high hypoxia, scarce nutrients, and very often therapeutic stress conditions. It was reported that in advanced HCC, autophagy has an oncogenic (prosurvival) role observed as an increased LC3-II expression that positively correlates with malignant progression and poor prognosis [125]. However, one should be careful when making conclusions regarding the influence of LC3-II overexpression on autophagic activity. Hence, LC3-II overexpression cannot be solely used as a marker for increased autophagic activity since LC3-II increased expression might have resulted from accumulation due to autophagy inhibition at the postlipidation stage. Specific hepatocyte Atg5 knockout mice revealed the tumor promoter role of autophagy in hepatocarcinogenesis [126]. Thus, the Atg5 ablation resulted with impaired autophagy in the liver and the development of benign hepatic tumors with no hepatocellular carcinoma. This inability to develop hepatocellular carcinoma was correlated with the induction of tumor suppressors, such as p53, p16, p21, and p27, which negatively regulated the progression of tumorigenesis when autophagy was impaired. Hence, autophagy in advanced HCC may block antitumor effects of tumor suppressors, and blocking autophagy altogether may be a promising target for the therapy of established HCC.

Since autophagy plays a dual role in the pathophysiology of malignant liver diseases, it is important to define whether a specific cancer type requires autophagy induction or inhibition to propagate. Back in 1985, Schwarze and Seglen were the first to indicate that autophagy acts to suppress liver carcinogenesis and HCC growth by limiting the cellular protein accumulation rate [65]. In 1993, Kisen et al. observed reduced autophagic activity in HCC cells and their precursors compared to normal hepatocytes [127]. This confirmed the hypothesis that reduced autophagy may be an important aspect of growth deregulation in liver cancer. Rapamycin and its derivatives act as autophagy inducers by inhibiting mTOR pathway [128]. In clinical trials, with the introduction of rapamycin, antitumor effect was shown along with improved overall survival rates in post liver transplantation patients with HCC (NCT00328770, Table 1) [129]. A similar effect was observed after using sirolimus in patients with advanced HCC who did not have a liver transplantation (NCT01079767, Table 1) [130]. This needs to be highlighted because it indicates that autophagy activation, and not only inhibition, may be beneficial in the HCC treatment. Unfortunately, everolimus, a rapamycin derivate, did not give such promising results and conflicting data have been obtained. Everolimus inhibits tumor growth in xenograft models of human hepatocellular carcinoma [131]. However, the later clinical phase III trial, EVOLVE-1, performed on patients with progressed HCC after sorafenib treatment or on patients that showed intolerance to sorafenib, did not show a significant difference in the overall survival rate when everolimus was administered (NCT01035229, Table 1) [132]. To that end, cell line experiments using a combination of different autophagic pathway targets indicate that this could be a promising therapeutic strategy in HCC treatment [133, 134]. For example, the synergistic effect of targeting mTOR with a combination of everolimus and another new-generation phosphatidylinositol 3-kinase/mTOR adenosine triphosphate-site competitive inhibitor BEZ235 or with the ULK1 inhibitor [133] suppresses proliferation of tumor cells.

Sorafenib, a multityrosine kinase inhibitor of mTOR pathway, an FDA-approved drug that induces autophagy, is also used for the advanced HCC treatment [135, 136]. Preclinical studies and several clinical trials confirm that sorafenib improves the survival of patients with advanced HCC, independently (NCT00105443, NCT00492752; Table 1) [136, 137] or in coadministration with other small-molecule drugs that inhibit HCC growth through autophagy induction [138, 139]. The problem with sorafenib-induced autophagy is a possible drug resistance in patients with HCC [140]. Therefore, further research is necessary to clarify the role of an autophagy-induced therapy approach in the treatment of HCC. Moreover, autophagy inhibition could also be a potential therapeutic strategy for HCC treatment, because autophagy is required for HCC cell survival, especially in the early stages [141]. Although there are about fifty ongoing clinical trials based on targeting autophagy for cancer treatment, only two current clinical trials are focused on autophagy inhibition using HCQ in HCC. The first one, a phase II trial, is studying if sorafenib-/HCQ-induced autophagy in HCC will have improved efficacy when compared to sorafenib treatment alone and if the addition to HCQ would lead to disease stability in patients with advanced HCC (NCT03037437, Table 1). The second clinical trial is in the recruiting phase I/II focusing on autophagy inhibition using HCQ in unresectable HCC (NCT02013778, Table 1).

### 4.6. Colorectal Cancer

Colorectal cancer (CRC) is the third major cause of cancer deaths in both sexes worldwide with the relative 5-year survival rate ranging around 65% after being diagnosed [59]. A complex multifactorial etiology of CRC is known, and, in recent years, autophagy has been recognized as one of the molecular mechanisms that regulate malignant transformation of CRC cells. Moreover, successful autophagy modulation, in several studies, proved to be very promising as CRC therapy.

The autophagy machinery provides multiple genes involved in the switch from normal to colorectal pathology. The first link between autophagy and CRC was discovered when autophagosomal marker, LC3-II protein, was found overexpressed in an advanced CRC compared to normal surrounding tissue [142, 143] suggesting that altered LC3 expression levels could indicate autophagy involvement in cancer. Also, low LC3 level has been linked to a better therapeutic response and prognosis in patients with advanced CRC [144, 145] suggesting a possible role of LC3 protein as a prognostic CRC marker. The prosurvival role of autophagy was suggested on the CRC cell line models after treatment with autophagy inhibitors, 3-methyladenine (3-MA) or CQ in combination with 5-fluorouracil (5-FU) and radiation therapy [77, 146].

Current knowledge on the role of Beclin1 gene in colorectal cancer is quite controversial since different studies show the opposite roles of Beclin1 in CRC carcinogenesis. Studies are reporting that Beclin1 overexpression can support tumorigenesis [147] but may also inhibit CRC cell growth [148]. This controversy is particularly emphasized when the survival prognosis is correlated with the Beclin1 expression and CRC. Both Zhang et al. and Ahn et al. have shown high Beclin1 expression in colorectal carcinoma tissue compared to healthy mucosa [147, 149]. Additionally, Ahn et al. studied this high Beclin1 expression in respect to invasion, metastasis, and cancer stage. However, no significant association of Beclin1 expression with clinicopathologic characteristics was reported [149]. Interestingly, another study analyzing Beclin1 expression in CRC patients has shown that those patients with a high Beclin1 expression had a better chance of being disease-free and had a better overall survival rate as compared to those with lower Beclin1 expression, indicating that high Beclin1 expression could serve as a favorable prognostic marker in CRC [150]. The controversial role of Beclin1 in CRC carcinogenesis was demonstrated by the results of two contradictory studies that tried to explain the connection between Beclin1 expression with a final clinical outcome in CRC patients who received 5-FU-based adjuvant therapy after resection. The first study showed that an increased expression of BECN1 is connected with a better clinical prognosis in patients who received 5-FU chemotherapy 6 months after resection [151]. The second study reported that Beclin1 overexpression was associated with reduced survival in CRC patients treated with 5-FU indicating a role for autophagy in drug resistance [152]. Further, low Beclin1 expression in patients with advanced CRC treated with cetuximab has been connected with longer disease-free survival [144] while later studies from 2014 to 2015 suggested a low Beclin1 expression as a prognostic biomarker for poor final clinical outcome [145, 153]. With this in mind, the role of Beclin1 in CRC still represents a complex puzzle in need of more extensive research.

Alterations in other core autophagy machinery components have also been associated with CRC and its progression and may potentially be good prognostic indicators. Mutations and reduced expression of Atg5 were found in many gastrointestinal carcinomas including CRC [154], suggesting the tumor suppressor role of autophagy in CRC. Unlike Atg5, the expression of Atg10, important in the elongation of the autophagosomal membrane, was increased in CRC and associated with invasiveness and generally worse prognosis [155].

Currently, there are several preclinical studies on CRC cell lines or mouse models using autophagy inhibitor CQ in combination with other agents such as 5-FU [156], histone deacetylase inhibitor—vorinostat [157], or proteasomal inhibitor—bortezomib [158]. It was shown that more successful effects are achieved with combined therapy than with monotherapy in respect to tumor growth reduction.

To conclude, the role of autophagy in CRC is still unclear and future studies will need to consider particular carcinomas individually to develop personalized anticancer therapy, which would take into account the autophagy status and its specific molecular changes.

### 4.7. Lung Cancer

The highest mortality rates of all cancer types belongs to lung cancer [59]. The lack of obvious symptoms in the early stages of the disease greatly postpones the diagnosis, which unfavorably affects the outcome of the disease. There are two major subgroups of lung cancer classified according to their histological appearance, small-cell lung cancer (SCLC) and non-small-cell lung cancer (NSCLC). The NSCLC is the most common type accountable for 85% of all lung cancer diagnosis [159]. Currently, the genetically engineered mouse models whereby the NSCLC initiation and progression are driven by the oncogenic KRAS or BRAF mutations are used to study the molecular aspects of the disease [159].

Previous studies on different cancer cell lines bearing activating mutations in Ras have shown that the autophagy level is higher than in the healthy cells suggesting that these tumors are autophagy-dependent and that autophagy could serve as a potential therapeutic target in NSCLC treatment [116, 160]. When the Atg7 was deleted in the mice lungs bearing NSCLC, a suppression of the tumor cell proliferation was noticed [116, 160]. These experiments have also shown that NSCLC cells with impaired autophagy accumulate morphologically abnormal mitochondria indicating that intact mitochondrial function is important for the growth and malignancy of NSCLC. It was also shown that due to impaired respiration and oxidation of the fatty acids, these tumors have a tendency to accumulate lipids and are more prone to starvation. Similarly, the Atg7 deficiency in mice with BRAF-induced lung tumor also resulted in accumulation of dysfunctional mitochondria ultimately leading to tumor growth restriction [161]. But one interesting difference between KRAS- and BRAF-driven NSCLC was also observed that relates to the mice overall survival. BRAF mutants with Atg7 deletion tend to live longer as opposed to mice with KRAS mutation that die from pneumonia instead of cancer suggesting that autophagy deficiency might promote inflammation [162].

Understanding of the exact mechanism by which autophagy promotes the NSCLC growth and progression would open the door to prospective therapy. To this end, Strohecker et al. have shown that in Atg7-deficient KRAS- or BRAF-driven NSCLC, addition of glutamine rescues the tumor progression suggesting that autophagy provides amino acids needed to fuel tricarboxylic acid cycle (TCA) [160, 161]. Therefore, it can be concluded that autophagy promotes malignancy through maintenance of the mitochondrial function in such a manner that it ensures availability of the mitochondrial substrates crucial for preventing energy deprivation. These studies have opened up a possibility to control KRAS- or BRAF-driven NSCLC growth by specifically modulating autophagy.

In addition, Zou et al. have shown that some NSCLC cells have elevated levels of autophagy as a consequence of the treatment with EGFR tyrosine kinase inhibitors (TKI) [163]. It was speculated that the observed upregulation of autophagy could be an alternative mechanism that promotes tumor cell survival in NSCLC cells resistant to the EGFR-TKI treatment. Therefore, targeting autophagy in combination with EGFR-TKI seemed a good treatment option that could overcome the resistance and enhance antitumor effect of these drugs. Indeed, two studies have shown that when an autophagy inhibitor CQ is used in combination with EGFR-TKI, NSCLC cells are more prone to respond to treatment either by overcoming the resistance in the wild-type EGFR NSCLC or by overcoming the antagonistic effect of EGFR-TKIs and therapeutic agents in wild-type and mutant EGFR NSCLC [163, 164].

A chemotherapeutic drug paclitaxel, also used in NSCLC treatment, was shown to be ineffective in some NSCLC. Apparently, paclitaxel induces the autophagy through decreased miR-216b levels that normaly downregulate Beclin1 activation and causes autophagy activation in paclitaxel-treated cells resulting in a decreased paclitaxel-induced cell death due to the activation of autophagic cancer cell survival [165]. Along similar lines, other preclinical studies conducted on NSCLC cells investigated the effect of the hormonally active form of vitamin D (1,25-D3) and vitamin D analogue (EB1089) in combination with radiation. It was found that EB1089 induces a novel cytostatic form of autophagy suppressing NSCLC proliferation [166]. Additionally, Zhang et al. have shown that simultaneous targeting of CD47 and autophagy in NSCLC xenograft models enhance antitumor activity through the activation of caspase-3, recruitment of macrophages, and overproduction of ROS [167].

Up until now, there have only been two clinical studies investigating the effectiveness of HCQ in combination with EGFR-TKIs in NSCLC treatment. One such study, published in 2012, was conducted to explore the safety, maximal dose tolerated, clinical response, and pharmacokinetics of HCQ with or without EGFR-TKI inhibitor, erlotinib [168]. This study has shown that HCQ is generally well tolerated and safe in NSCLC patients previously treated with EGFR-TKIs. However, low response rates observed in the study cohort were assigned to potentially ineffective doses of HCQ or by heavily pretreated patient populations (NCT01026844, Table 1) [168]. The other currently ongoing study is investigating erlotinib with or without HCQ in chemo-naive advanced NSCLC (NCT00977470, Table 1).

Overall, the clinical data on autophagy inhibition in NSCLC patients is scarce but promising, suggesting that further work is needed to fully elucidate the possibilities of autophagy inhibition especially in patients with NSCLC resistant to EGFR-TKIs.

## 5. Conclusions and Future Perspective

The role of autophagy in cancer is enormously complex, and our knowledge in this field is currently very limited. Deciphering the autophagy in context of tumor complexity and heterogeneity is necessary to fully understand their complex and intertwined association.

Despite the seemingly paradoxical and dual role of autophagy in the context of tumor initiation and development, studies on cellular and mouse models have confirmed that there are two main principles of autophagy actions in tumors. Autophagy-deficient mouse models demonstrate that at the beginning of tumor development, basal autophagy is generally able to inhibit tumor formation by suppressing the DNA damage and genome instability. However, in contrast to this suppressive role, autophagy facilitates tumor progression in most cancers. Does autophagy always serve as a mechanism for providing energy and nutrients in developing cancers or is this theory only limited to some cancers? Most of the current studies confirm that cancers use autophagy to obtain nutrients needed to sustain tumor growth, but the question is why autophagy suppressor-based therapies are often not effective enough? Our understanding of what autophagy specifically does at the molecular level and how it influences different tissues, tumors, and genes is currently very limited. Albeit, there is expanding knowledge from preclinical studies and clinical trials on the possible utilization of autophagy as an anticancer immunotherapy. The first results indicate that optimal combination of autophagy inducers or inhibitors with chemotherapy is going to be important approaches for even more successful therapies (summarized by Pan et al., *Oncotarget*) [169]. Additionally, do we know which type of autophagy contributes to the development of a single tumor and could selective autophagy respond to the question of unsuccessful autophagy suppressor-based therapy of cancer? To better understand the role of autophagy in tumors, additional basic research is needed in the field of molecular biology, biochemistry, and chemistry but above all in molecular oncology.

According to the latest data, 53 out of 81 international clinical studies investigating autophagy modulation as a possible target in disease therapy are focusing on cancer. Based on the fact that about 70% of clinical studies are focusing on autophagy role in cancer indicates that the potential of autophagy modulation in cancer treatment is promising. Clinical trials involving autophagy modulation in cancers have been designed to assess the effect of autophagy inhibition in combination with other conventional therapies. Only a minority of clinical trials on lung, glioblastoma, pancreatic, melanoma, breast, and prostate cancers are testing CQ/HCQ as a monotherapy. This is most likely due to the fact that we currently do not have good autophagy-specific modifiers as well as due to cancer complexity where it is almost exclusively needed to target more than one cellular pathway to generate a successful therapy. Therefore, it is important to encourage chemists and pharmacologist to design and synthesize novel and highly specific autophagy modulators and to further engage cellular and molecular biologists, computational biologists, and mathematicians in pharmaceutical industry and academia to a collaborative network.

The opened question remains, why combined therapy is almost always more effective than monotherapy and what are the consequences for using this aggressive combination? Based on preclinical animal studies, so far there are not many reported side effects of autophagy inhibition such as cancer remission, appearance of secondary tumors, metabolic disorders, or infections (except potential induction of inflammation in case of autophagy deficiency mentioned earlier). Moreover, there is no available data considering the consequences of other molecular pathway inhibitions that are used in combined therapy. Unfortunately, we still lack the knowledge and tools to specifically activate or inactivate autophagy without disturbing other cellular processes and to specifically modulate autophagy within the tumor cells concurrently avoiding autophagy disruption in healthy cells.

A major problem with the clinical trials is how to identify patients who are most likely to benefit from autophagy-based cancer therapy. To estimate if autophagy modulation is an effective therapy, the critical point would be to measure the autophagic flux *in vivo*. Currently available biomarkers for monitoring autophagy flux in clinical trials consist of tracking the accumulation of autophagic vesicles in tumor cells as well as monitoring the status of LC3 lipidation by Western blotting or immunohistochemistry. The basic research scientists are at the moment less limited with the tools and resources necessary for the complete autophagy assessment in different settings and conditions. However, much more *in vivo* experimentation is needed for new or improved techniques, so taken together it makes future clinical applications more difficult and challenging.

What is well known is that disturbed autophagy is the basis for many diseases including cancer. To date, translating preclinical knowledge of autophagy modulation into clinic has progressed rapidly in the field of oncology. Although there are very few reported clinical results confirming the effectiveness of autophagy modulation in cancer, in several other pathologies, such as obesity and diabetes, cardiology, neurobiology, and immunology, preclinical studies give promising results. Therefore, it is a huge challenge and task for both, scientists and clinicians, to better understand the molecular mechanisms of autophagy and carcinogenesis in order to successfully translate preclinical knowledge to a clinical environment. Also, the development of new autophagic modulators, activators or inhibitors, is necessary to selectively target the newly discovered autophagic signaling molecules that represent the key players in both canonical and alternative autophagy pathways. Above all, it will be of great importance to access the individual approach to cancer and its autophagy status.

## Figures and Tables

**Table 1:** Autophagy modulators in preclinical studies and clinical trials for cancer therapy.

	Cancer type	Autophagy modulators	Mode of action	Model tested/clinical trial phase	Reference of study/trial reference at ClinicalTrials.gov
Pancreas cancers	PDAC	CQ	Lysosomal inhibitor	Pancreatic cancer xenografts and mouse models	[100]
	PDAC	HCQ	Lysosomal inhibitor	PDAC mouse model	[101]
	Stage IIb or III pancreatic adenocarcinoma	HCQ + gemcitabine	Lysosomal inhibitor + inhibitor of DNA synthesis	Phase I/II	NCT01128296
	Advanced and metastatic pancreatic adenocarcinoma	HCQ + gemcitabine/abraxane	Lysosomal inhibitor + inhibitor of DNA synthesis/cell division inhibitor	Phase I/II	NCT01506973
	Metastatic pancreatic adenocarcinoma	HCQ	Lysosomal inhibitor	Phase II	[102]/NCT01273805
	Resectable pancreatic cancer	HCQ + capecitabine	Lysosomal inhibitor + inhibitor of DNA synthesis	Phase II	NCT01494155
	Resectable pancreatic adenocarcinoma	HCQ + gemcitabine + nab-paclitaxel	Lysosomal inhibitor + inhibitor of DNA synthesis + cell division inhibitor	Phase II	NCT01978184
	Resectable pancreatic adenocarcinoma	Gemcitabine, nab-paclitaxel, HCQ +/− avelumab	Inhibitor of DNA synthesis + cell division inhibitor + lysosomal inhibitor +/− T-cell activator	Phase II	NCT03344172

Breast cancers	Metastatic breast cancer	HCQ + ixabepilone	Lysosomal inhibitor + cell division inhibitor	Phase I/II	NCT00765765
	Advanced or metastatic breast cancer	CQ + taxane	Lysosomal inhibitor + cell division inhibitor	Phase II	NCT01446016
	Ductal carcinoma in situ	CQ	Lysosomal inhibitor	Phase I/II	NCT01023477
	Primary invasive breast cancer	CQ	Lysosomal inhibitor	Phase II	NCT02333890

Hepatocellular cancers	HCC after liver transplantation	Sirolimus	mTORC1 inhibitor	Phase II/III	[125]/NCT00328770
	Advanced HCC	Sirolimus	mTORC1 inhibitor	Phase II	[126]/NCT01079767
	HCC	RAD001 (everolimus)	mTORC1 inhibitor	Xenograft models of human HCC	[127]
	Advanced HCC	RAD001 (everolimus)	mTORC1 inhibitor	Phase III	[128]/NCT01035229
	HCC	RAD001 (everolimus) + BEZ235	mTORC1 inhibitor + new-generation mTORC1 (mTORC2) inhibitor	Human HCC cell lines and HCC mouse model	[130]
	HCC	RAD001 (everolimus) + SBI-0206965	mTORC1 inhibitor + ULK1 inhibitor	Different human cancer cell lines	[129]
	Advanced HCC	Sorafenib	mTORC1 inhibitor	Phase III	[132]/NCT00105443
	Advanced HCC	Sorafenib	mTORC1 inhibitor	Phase III	[133]/NCT00492752
	HCC	Sorafenib and SC-59	mTORC1 inhibitors	Human HCC cell lines and HCC mouse models	[134]
	HCC	Sorafenib + pemetrexed	mTORC1 inhibitors	Different human cancer cell lines and mouse models	[135]
	Advanced HCC	Sorafenib + HCQ	mTORC1 inhibitor + lysosomal inhibitor	Phase II	NCT03037437
	Unresectable HCC	HCQ	Lysosomal inhibitor	Phase I/II	NCT02013778

Colorectal cancers	CRC	CQ + 5-FU	Lysosomal inhibitor + inhibitor of DNA synthesis	CRC cell lines	[142, 150]
	CRC	3-MA + 5-FU	Lysosomal inhibitor + inhibitor of DNA synthesis	CRC cell lines	[77]
	CRC	CQ + 5-FU	Lysosomal inhibitor + inhibitor of DNA synthesis	CRC cell lines + mouse models	[150]
	CRC	CQ + vorinostat	Lysosomal inhibitor + histone deacetylase inhibitor	CRC cell lines + mouse models	[151]
	CRC	CQ + bortezomib	Lysosomal inhibitor + proteasomal inhibitor	CRC cell lines + mouse models	[152]

Lung cancers	NSCLC	CQ + erlotinib	Lysosomal inhibitor + EGFR-TKI	NSCLC cell lines	[157]
	NSCLC	CQ + gefitinib + cisplatin	Lysosomal inhibitor + EGFR-TKI + inhibitor of DNA synthesis	NSCLC cell lines	[158]
	NSCLC	CQ + SIRP*α*D1-Fc	Lysosomal inhibitor + cell division inhibitor	NSCLC cell lines	[161]
	Advanced NSCLC	HCQ	Lysosomal inhibitor	Phase I	[162]/NCT01026844
	Advanced NSCLC	HCQ + erlotinib	Lysosomal inhibitor + EGFR-TKI	Phase I	[162]/NCT01026844
	Advanced NSCLC	HCQ + erlotinib	Lysosomal inhibitor + EGFR-TKI	Phase II	NCT00977470

PDAC: pancreatic ductal adenocarcinoma; HCC: hepatocellular carcinoma; CRC: colorectal cancer; NSCLC: non-small cell lung cancer; CQ: chloroquine; HCQ: hydroxychloroquine; 5-FU: 5-fluorouracil; 3-MA: 3-methyladenine; SIRP*α*D1-Fc: signal regulatory protein *α* D1-Fc; mTORC: mammalian target of rapamycin complex; ULK1: Unc-51-like kinase 1; EGFR-TKI: epidermal growth factor receptor-tyrosine kinase inhibitor.
